# Supplementary material for: Biomedical open source software: Crucial packages and hidden heroes
Source: PLoS Comput Biol. 2026 May 13;22(5):e1014260. doi: 10.1371/journal.pcbi.1014260 (PMC13235917; doi:10.1371/journal.pcbi.1014260)
Supplement: Attachment — Submitted filename: rebuttal.pdf [file pcbi.1014260.s002.pdf]

Answers to reviewers

We are grateful to the reviewers for their sharp and well-thought-out comments. These comments led to many rewrites. Below we try to answer them item by item. Some comments suggested ideas for future research, and we are thankful for many useful insights. We rewrote many parts of the paper and hope it is much clearer now.

Reviewer #1

> **1. The authors justify the use of the Katz centrality metric as fulfilling some basic criteria, but other than excluding two related metrics (eigencentrality and PageRank) the argument for Katz centrality is not very well developed, neither from a theoretical (e.g., deriving from a set of axioms) or from a purely empirical perspective.**

We extended the discussion of centrality measures and further explained why Katz centrality corresponds to our intuition about the importance of the node for science and technology:

> We found that Katz centrality Katz [1953] satisfies all our criteria. Katz centrality can be interpreted as the importance of the node for the diffusion processes on the network. This corresponds to the intuition about innovation being a diffusion-like process Rogers [2003]. Katz centrality gives us the attenuation factor β as a free parameter to control the importance of papers (which we set equal to 1), such that paper nodes contribute a factor proportional to β times their citation count to the software packages they mention (we follow the formulation of Katz centrality in Sun and Tang [2011]).

> **2. The authors write that "We found that Katz centrality [Katz, 1953] satisfies all our criteria. This approach gives us the attenuation factor β as a free parameter to control the importance of papers..." This requires more detail. The original [Katz, 1953] paper does not mention a parameter β (in fact, it is typeset without the use of Greek letters, and mentions only the attenuation factor a, which today we would typeset as α). The authors should clarify what they mean by this and present the actual formulas they use. Is β the attenuation factor? It does not seem to be the case as it would not be related to the importance of papers (at least in my understanding, but I may be wrong given the lack of detail in the manuscript).**

We added a quotation for a more modern formulation [Sun and Tang, 2011] where the role of the parameter β is better explained.

3. The authors acknowledge that the "velvet" is a misassignment. The velvet assembler is hardly under-appreciated (Google Scholar gives it >11,000 citations: `https://genome.cshlp.org/content/18/5/821.short`, `https://scholar.google.com/scholar?cites=355244555212756650`**). Having had a look at their dataset, it seems that the highly cited paper mentioned in the text appears to be** `10.1093/bioinformatics/btu170` **(the Trimmomatic paper) which is marked as citing "velvet", but reading that paper, it cites the velvet assembler, not the velvet package on PyPI.**

Without this example, there are no other examples of under-appreciated tools in the manuscript, which was a centrally motivating factor for the study. In fact, from Figure 3, I would even be tempted to say that citations do a good job of capturing the centrality of tools, although it is also difficult to say because this figure does not provide a lot of detail for the papers with <5000 citations (as the dots overlap and are impossible to distinguish), which constitute the majority of papers.

This was indeed a bad example. We completely rewrote this part, added tables of packages in different quadrants and the discussion of this package and others in the "Nebraska" category. We are grateful for this remark since it gave us an idea how to make the paper better.

4. The code is available, but references paths such as `~/Downloads/pruned-network.csv` **which are not directly usable.**

We have updated the repository, ensuring all computations are reproducible, and adding instructions to reproduce our work in the README.

Reviewer #2

While this work has a lot of potential, the manuscript suffers from a serious lack of tangible findings that would confirm the validity of the approach. The results consist in a dense graph from which users cannot draw conclusions, and the name of 9-16 packages identified as the most "central" given the authors' metrics. There is little to no investigation of these packages, which are in any case far too few to allow the authors to conclude anything about the approach. The only package for which some scrutiny has been applied is held as a new-found critical package that is hardly mentioned in the literature (velvet), but the authors themselves cast shadows on the veracity of that finding since it only appears because its original method

is historically important (see first major comment). The authors identify and clearly state many interesting limitations to their approach (which is laudable), whether from a consequence of imperfect data or of their metrics itself. But they do not attempt to quantify how much these limitations are impacting their measure through validation of the prominent packages or known/expected packages (see major comments 2 and 3). Finally, I find the manuscript difficult to parse and suggest several axis of improvements, as well as additional information that would make it easier for readers to judge the method.

We rewrote the discussion, adding tables of packages in different quadrants of the diagram and a discussion of the findings.

> **1. One of the main results (if not the only one that is actually discussed) of the paper is the identification of "velvet" as a "Nebraskan" package. Two aspects of that result bother me: firstly in the way it is introduced, and secondly, in what it actually says about the method. In the discussion section, "velvet" is first introduced as the only (for lack of another one mentioned) "Nebraskan" package, and the authors then state that the package disappears as soon as the paper citation counts are not taken into account (unweighted graph). In another section of the discussion, they double down on by highlighting again that it is mostly, if not only, central because of one highly cited paper (presumably the original publication).**
>
> **In my opinion, this totally undermines the method, which aims at identifying specifically "Nebraskan" packages. Indeed, the only identified "Nebraskan" package only pops in the analysis because the original method is highly cited (11000 citations)... While I cannot exclude that the package is still used (there are a few issues from 2022 in the Github repository), it clearly is not a dependency of other software (from the author's own analysis), it is 17 years old (last commit 11 years ago) and the paper is probably cited because of its historical importance. A quick question to genomics specialist made it clear that newer tools are more useful nowadays: Abyss, PacBio, Nanopore. Genomics being such an active field, I have a hard time believing that velvet is still crucial to the scientific infrastructure.**
>
> **To justify the power of the method, better (so far unknown) examples should be highlighted to the readership.**

This is a valid critique. We rewrote the *Results* and *Discussion* section, adding tables of notable packages and discussing the findings.

2. As exemplified by velvet, the manuscript lacks analysis validating the method. Only 9 (fig 2, or 16 in fig 3) packages are mentioned, of which a single one is investigated. I strongly suggest examining closely a non-negligible number of packages (e.g. 50-100, number should be adjusted based on how much work it ends up being, while maintaining enough statistics to be able to draw conclusions) that fall into the "Nebraskan" and "Pasteur" categories (given ad-hoc thresholds on mentions and centrality) and identifying the amount of "potential impostors" (e.g. like velvet) in that pool.

The authors should go through the list of identified packages and compile metrics and metadata information to validate whether the package could belong to the assigned category:

- **Is it an homonym?**
- **Centrality in weighted vs unweighted graphs**
- **Number of mentions in the CZI dataset**
- **Download statistics (which are available in all three package ecosystems investigated)**
- **Average citation / paper with edges to this package, highest citation count for paper with edge (aka guarding against velvet-like packages)**
- **Date of the latest commit in Github / Date of the latest package version**
- **Rate of updates (e.g. average commits / months in the last year or versions / year in the last 5 years)**
- **Number of Github stars if applicable**
- **...**

None of these are perfect proxy metrics (e.g. download statistics will not be comparable between ecosystems, CI/CD can skew the downloads etc.), but they can all inform to a certain extent on whether there might be issues with the particular software packages.

Additionally, assigning a software category to these packages would allow refining the analysis and help understand what type of packages the method is able to classify. I don't know if ontologies already exist, but categories could be along the lines of: specific analysis (BLAST, velvet), generic computation (scikit-learn), infrastructure / data structures (numpy, pytest, pandas), rendering (ggplot2, matplotlib).

The results could be show in tables, for instance per categories and per ecosystem, before being discussed.

3. An additional important experiment to validate the method is to build a list of known packages and inspect where they appear in the [Mentions, Centrality] graph. While the authors correctly state that different fields will assign importance to different packages, and such importance will indeed reflect mostly direct use of package (as opposed to usage through dependencies), this does not mean that one should not investigate what happens to clearly important scientific software package. Such a list can easily be built by surveying colleagues or asking in forums (biostars and image.sc already cover a large range of biomedical applications, missing mainly the medical fields). The authors could make sure that they are building a list that covers examples in categories spanning application of a particular analysis, generic analysis package, data i/o, rendering or more infrastructure ones. Examples that come to mind are for instance dplyr (I am limited in my knowledge of R), xgboost, pandas, matplotlib, scipy, scikit-learn, dask, pytorch, ggplot2 (obviously that one is already clearly identified in the current analysis, although it is assumed that the readers are familiar with it and it is not discussed). The centrality and mentions measures could be reported for these packages per ecosystem and discussed in comparisons to those identified via thresholding the metrics (Nebraskan and Pasteur), as well as some of the other metrics.

The validity of the analysis mostly depends on the software disambiguation of CZ Software Mentions dataset Istrate et al. [2022], which was discussed in the original publication and analyzed in the subsequent works. The application of this analysis to the robustness of the centrality calculations is a very interesting research program. While it is rather out of scope for this publication, we are planning to implement it.

- **The Stokes-like diagram is central in the introduction, without describing what a "Pasteur" package is, forcing readers to google the Stokes diagram. "Nebraskan" and "Pasteur" are mentioned in the text a lot, without exemplifying it in the result figures.**

We added the discussion of the original Stokes ideas to give the context to the reader.

- **Mentioned software packages should be identified with references and links to the package as well as with a short description of what they are. Otherwise, readers are forced to investigate on their own what these packages are (e.g. I had to google velvet, and until I**

read the discussion I had no idea why this package was picked up by the analysis at all).

We added references and descriptions to the packages mentioned.

- **Discussion about the packages are done piece-wise in multiple places and make it difficult to follow the logic regarding the package (velvet).**
- **A substantial part of the discussion is actually results (metrics for velvet, appearance and its disappearance in the different graphs, package coverage).**

We rewrote the discussion part of the paper to address the issues raised by the reviewer.

5. The authors state that they are using the CZI Software Mentions Dataset in the introduction, but do not describe it. Readers have to wait for the discussion to learn about potential pitfalls (cluster of names, homonyms) that may impede the analysis. In addition, the authors should add a few numbers to highlight the importance of the dataset: e.g. number of papers and number of software in the dataset.

We added a short discussion of the dataset and cited the paper discussing its limitations in more detail.

6. As opposed to what is stated in multiple places (e.g. point 9 in the discussion) in the paper, PyPI is not a package manager, but a package repository (same for CRAN?). pip/conda are indeed package managers for the Python ecosystem.

We changed the terminology using a more appropriate word "ecosystem".

7. First paragraph introduction: "This is true not only for the sciences", prefer the term "natural sciences" to not appear derogatory to sociology.

A good comment. We rephrased this statement.

8. Paragraph 2 and 4 in introduction repeat each other.

We rewrote the text to avoid redundancy and improve the flow of reading.

9. The authors state that "We also found less central packages mentioned heavily by specific communities (e.g. PRISMA in CRAN [. . .]", but it seems to me that PRISMA is the most central package identified by their method (Fig 3) solely based on the metrics.

We changed the wording to show that certain packages are specific to certain communities.

10. The authors should mention in the result sections how many software packages are identified through the dependency trees, that is the total number of software packages in their graph.

We are grateful to the reviewer for this great suggestion. We added the corresponding table and discussion to the paper.

11. The result section or the supplementary materials should provide distributions of centrality and of mentions, as well as averages. A comparison of how the distribution of centrality measures changes between unweighted and weighted would be interesting.

We agree this is an interesting question. We added a discussion of summary statistics and Gini coefficients of the distributions.

12. Could the authors discuss the largest connected component? How do they interpret it? How come it seems to be cross-ecosystem? If a paper mentions both python and R package, does that make their dependency tree a single connected component?

We are grateful to the reviewer for showing us the misleading writing on our part. The largest connected component was calculated separately for each ecosystem, and we have made this clearer in the paper.

13. I tried running the Github example notebooks, but these failed due to json errors or missing json (e.g. `pypi_with_mentions.ndjson`). I have not investigated further. I would suggest making sure that the notebooks run, and that the README explains clearly how to get the data, run the analysis and explore the results in order to benefit the readership!

We have updated the repository, ensuring all computations are reproducible, and adding instructions to reproduce our work in the README.

1. It would be nice to add explanations about how Katz centrality is calculated in your analysis in the supplementary materials of the paper.

We added the discussion of the calculation of Katz centrality to the paper.

2. The authors noted that "we found that roughly 10% of software packages are part of dependency loops". I am a bit confused by this, do they expect these to be working software? Can such software build in the R and Python world?

The dependency we discuss is the metadata dependency. A loop means that installation of package A requires the installation of package B, *and* the installation of package B requires the installation of package A. Modern package managers resolve this loop by always installing these packages together.

Interesting enough, even run-time dependency loops are not fatal. Consider a Python package `foo` with one line

```
import bar
```

and a package `bar` with one line

```
import foo
```

Importing either package does not create endless loop, because Python keeps track of imported packages and does not import them twice. The same is true for R libraries, LaTeX packages and virtually all modern languages. Thus a dependency loop, while often indicating sloppy software practices, is not a show stopper.

We added more discussion regarding these cycles to the paper.

3. When the authors state that "It suggests a more robust design structure in the universe of scientific software than in the general software world.", I would precise "open source software world" as company code / closed source software are often better organized due to internal constraints. There are other mentions of this general software world.

We agree with this suggestion and have added this qualification to the text.

4. When the authors state that "The lack of cycles also improves our analysis with Katz centrality, as it excludes feedback mechanisms that can artificially inflate the centrality of packages on a loop", do they mean that the lack of cycles exclude feedback mechanisms? This makes sense to me, but on the first read I had the impression that the Katz centrality calculation excluded feedback mechanisms.

We reworded this passage to make it clearer.

5. It is hinted at, but worth insisting on for the readership that, at a time where research institutions have a hard time battling constant cybersecurity targeting, open source software are potential entry ways for malicious actors (similar to the XZ Utils case).

We did mention the XZ Utils incident, but we agree with the reviewer that this case could be made stronger. We added a sentence to this effect in the Introduction.

Reviewer #3

- **It would have been interesting to approach leaders in the CRAN, PyPi and Bioconductor community for their views in the results - are there surprises or is it confirming intuition?**

This is a very good suggestion. We are discussing the findings with the community—and hope the publication will help with making them more visible.

- **The lack of dependency cycles is suggested as robust design - is it just less complexity or more homogeneity in the scientific software world? Or less legacy? Or smaller well connected development communities?**

These are very good questions. To tell the truth, we do not know the answers. The practice of scientific software writing surely deserves a deep research, and our paper is just one small contribution here.

- **Figure 3 the weighted complete graph and the LCC cluster pattern is identical (as could be expected) so it's unclear what the point is of showing this is.**

The results are indeed very close. This demonstrates the robustness of the estimate, thus we decided to show them both.

- **Figure 2 - apart from the labelled packages the rest of the figure is tricky to interpret. Unless it is further discussed its value is not clear**

We added tables to provide more insight in the result. Perhaps now the structure is clearer.

- **The paper focus was in three packages in each ecosystem. What about the next 10?**

We agree this is interesting. We added many more packages in three quadrants in the tables.

- **The bioscience literature community is interesting - there are dedicated journals for bioinformatics and others for scientific discovery that may mention the immediate software. Are there any differences in the citation (and hence weighting) pattern between different classes of journal?**

This is a very interesting question, which we hope to answer in the future work.

References

Ana-Maria Istrate, Donghui Li, Dario Taraborelli, Michaela Torkar, Boris Veytsman, and Ivana Williams. A large dataset of software mentions in the biomedical literature. *arXiv*, 2022. doi: 10.48550/ARXIV.2209.00693. URL `https://arxiv.org/abs/2209.00693`.

Leo Katz. A new status index derived from sociometric analysis. *Psychometrika*, 18(1):39–43, 1953. ISSN 0033-3123. doi: 10.1007/BF02289026.

E.M. Rogers. *Diffusion of Innovations, 5th Edition.* Free Press, 2003. ISBN 9780743258234.

Jimeng Sun and Jie Tang. A survey of models and algorithms for social influence analysis. In Charu C. Aggarwal, editor, *Social Network Data Analytics*, pages 177–214. Springer US, Boston, MA, 2011. ISBN 978-1-4419-8462-3. doi: 10.1007/978-1-4419-8462-3_7.
