# Supplementary material for: Biomedical open source software: Crucial packages and hidden heroes
Source: PLoS Comput Biol. 2026 May 13;22(5):e1014260. doi: 10.1371/journal.pcbi.1014260 (PMC13235917; doi:10.1371/journal.pcbi.1014260)
Supplement: Attachment — Submitted filename: ResponseToReviewers.pdf [file pcbi.1014260.s003.pdf]

Answers to reviewers

We are again grateful to the reviewers for their sharp and well-thought-out comments. Below we try to address these comments item by item and highlight changes to the manuscript.

> Reviewer #4: In this article, the authors combine two metrics (popularity and centrality) to identify specific classes of biomedical open source software. The authors rightly point out that software maintenance and funding is crucial to modern science. They add that their approach could be used to highlight the importance of some software packages and help their recognition and funding.
>
> The authors use software from three ecosystems. PyPI, Cran, and Bioconductor. This covers a lot of the bioinformatics / -omics tools, but does not cover much of the imaging software used by other part of the community. While this is a limitation, I think it is ok to start with a subset of biomedical research software and let other communities apply the method on their own field. The authors provide the software to do so.

We appreciate this comment, demonstrating the method to encourage discussion of appropriate metrics and build awareness of the datasets is part of the contribution we hope for from this paper.

> Disclaimer : while I am aware that the paper has been reviewed before, I was not in the first set of reviewers. The reviewers already covered important points.
>
> While this is an interesting proof of concept, It is hard to estimate this work because, as any metric, this metric is largely arbitrary. This does not make it uninsightful, but I am not currently convinced. I have some questions & concerns before being able to fully assess the manuscript :
>
> - Elephants in the room My main surprise is to not see cornerstone libraries such as Blas & Lapack, or even higher level libraries such as numpy / scikit learn appear in this graph (while scikit-learn appears in the mentions dataset). In a way, there is nothing more central than those libraries ; as far as I know, most python scientific libraries will depend on numpy, that uses Blas & Lapack. I am surprised that they do not show up in the analysis. Why is it so ? It is hard for me to trust the results knowing that.

We now address this directly in the limitations section on p13; it is caused by data limitations discussed for the next point.

> - Cutoff Where should the analysis stop ? In a way the code of the python/R interpreter, or of clang/gcc, is probably the most critical to this. Or even the windows OS and Linux Kernel ? Is there a cutoff somewhere ?

Indeed. We discuss this now directly on page 13. Our cuttoff is determined by our data-sources, which do not extend as deeply into the software stack as would be needed. We highlight this as a limitation and discuss possible approaches in the Discussion.

> - Anaconda vs Pypi Anaconda offers a very structured model for dependencies and distributing through conda-forge is much stricter on the dependencies than PyPI. Could looseness of the PyPi requirement system explain why obvious packages such as numpy/scipy/scikit lean do not appear ?

Indeed, we address this insight on Page 13; our datasources are limited in their ability to cross outside language communities in listing their dependencies. Anaconda, being cross-language for managing dependencies, may allow deeper traversal. BLAS, in particular, is often incorporated at lower levels than the packages that would be mentioned as dependencies even in Conda. But future work needed here.

> - The need for a metric : Unfortunately science is very much metric-driven. While I believe the authors propose this in good faith, metrics come at a price as they can become decision tools (which is implied by the authors when they mention the importance of recognition for funding) ; then often the metric itself is optimized, instead of what it should measure(Goodhart's law). I nontheless think that this work is interesting out of curiosity.

> - History vs current situation As the authors mention, software is a living environment. Some libraries will disappear and others will appear. It seems that the author's analysis give a lot of weight on history, as evidenced by velvet, but also tophat. Tophat has not been updated for many years, nor is being actively forked recently. Is it still actually a cornerstone ? More generally, how do you distinguish history from the current situation ?

We agree that change over time is crucially interesting. We address this with a new point in our discussion and point out this opportunity for future work.

> **Reviewer #5**: Biomedical open source software: Crucial packages and hidden heroes is well-written and interesting. It addresses a very important topic of recognition and credit/incentives for open-source scientific software used—and frequently developed in—the academic setting. The traditional transactional nature of academic collaboration and authorship does not capture well software development efforts, let alone software maintenance efforts. Package and library dependencies in particular are easily taken for granted and their developers and maintainers are unsung heroes. In particular, researchers may rarely mention how they loaded data or performed basic numerical calculations. Thus, the goal of this paper to develop robust methods and metrics that can account for their role is very commendable and very important.
>
> The traditional publishing side of academia has—for better or worse—the ubiquitous impact factors and h-indexes. Similar metrics for software contributions could help legitimize scientific software development efforts, particularly for early career researchers. I think these issues will only be further magnified both in the field of computational biology, but also in other scientific areas that may not been traditionally "computational", with the growth of machine learning and challenges of big data.
>
> To my knowledge, the approach here using Katz centrality is novel, not having been applied to the topic at hand. However, it is well motivated and explained in the text. While the dataset used is somewhat out of date at this point, this is not a major limitation, because the method should still provide insight into the dataset and then in the future be applied to the latest data. As a result, the work is of significant importance to the field and well suited for publication.

We appreciate these supportive comments.

> Major comments I'm not very familiar with CRAN and Bioconductor, but I am quite familiar with the scientific Python ecosystem. As a result, I must say that I am quite puzzled and somewhat troubled by the lack of core packages like numpy, scipy, and matplotlib figuring anywhere in the results. I fully expected to see them in the "Pasteur" quadrant. Just taking numpy as an example, pymol, a "Pasteur" package, has numpy as a dependency, as does pace, a "Popular" package. Whether a result of the specifics of the underlying data set or some aspect of the methodology, I think it's important that a package like numpy be accounted for somehow/somewhere in the text. Was

> it is simply considered a far outlier and as such discarded? Is it somehow a function of the age of the dataset (I have a hard time believing this)?

We investigated this in detail. These packages were in the expected locations in the analyses but a little lower down than our choices for labeling in the figures and showing the tables. We agree that locating these within the output is important for confidence of the reader and so now we highlight these packages (along with some important image packages like napari) in the figures, tables, and in the text.

We also provide the full output of the analysis as csv files in a GitHub repository to encourage readers to further inquiry without running the analysis software.

> With that said, I was impressed that the package tifffile was identified as a "Nebraska" package! It indeed is the most powerful package for loading tiff files in Python (that I know of), but libraries used to load data, like images, are unlikely to be mentioned in a publication. Further, tifffile is literally developed and maintained by a single person, although I believe they are in California and not Nebraska.

We agree that this is an exciting example of the potential of this kind of analysis; we further investigated the package and note this in the text.

> Minor comments - Page 3, The legend of Figure 1: it would be best to also have "Nebraska" and "Pasteur" quadrants briefly explained in the legend, to make the overall figure able to stand on it's own. "Nebraska" in particular is quite a logical leap, even for those familiar with the XKCD cartoon.

We now note this in Figure 1 caption.

> - Page 3, "Nebraska" packages: it would be nice to connect back to the XKCD cartoon as mentioned earlier. In the earlier context on Page 2, the emphasis is on "thanklessly" and I had glossed over the "Nebraska" part of the quote. Thus, even as someone familiar with the cartoon, I was briefly confused by "Nebraska" on page 3. Alternately, consider on Page 2 to say that the term "Nebraska" will be used for such packages/libraries.

Thanks. We now note this in text and in a footnote on Page 3.

> - Page 5, Results, a number of packages/libraries are mentioned here, but no references are provided, which is a bit ironic given

> the nature of the work. Some packages/libraries do get references later on, e.g. vctrs, reference 34, but I think all should be cited either in the text or in a table.

Indeed! This was an oversight that we regret; we are happy that reviewers are asking for these citations and we have provided many more now.

> This would also help fully disambiguate packages/libraries. For example, 'tophat' on PyPI is a mobile app framework from 2012, so I have some doubt it that's really the "Pasteur" package—perhaps it's 'top-hat' a "Recommendation system in TensorFlow"?

We investigated this in detail, and we now discuss this as an example of the challenges of package disambiguation. We cannot solve these, given the current dataset, so we are only able to discuss the issue, reason about the limits of our claims, and highlight future opportunities. See page 12.

> - Page 5, Results, "(a) unweighted graph, (b) weighted complete graph, and (c) the largest connected components of the weighted graph" — it would be helpful to clarify the differences between these three approaches. There is some interpretation of the differences in the Discussion, but here where these 3 models are first mentioned it would be helpful to clarify the differences in approach.

Thanks! We implemented this suggestion in section 2.2 before beginning to present our results.
