# Supplementary material for: Biomedical open source software: Crucial packages and hidden heroes
Source: PLoS Comput Biol. 2026 May 13;22(5):e1014260. doi: 10.1371/journal.pcbi.1014260 (PMC13235917; doi:10.1371/journal.pcbi.1014260)
Supplement: Attachment — Submitted filename: SoftwareDependenciesPaper_Response3rdround.pdf [file pcbi.1014260.s004.pdf]

Response to reviewers (3rd round)

Thank you for these reviews. We are pleased to see that each of the reviewers sees value in the paper and its results. While we can't make the research perfect we can acknowledge the issues and thus advance the field. We hope these changes make the paper acceptable.

> Reviewer #5: The Authors addressed my comments; however, after adding numpy, scipy, and napari to the analysis, they don't cite these packages when they appear in the text, e.g. page 12. Again, I find this very ironic given the nature of the work at hand. Please ensure that all of the packages discussed are cited properly!

Ironic indeed! Those mentions were added in the last round and we did not include citations. We now believe that we have cited all the software mentioned in the text of the paper (harder than it looks!). We do not directly cite the software names mentioned in tables and figures as we judge this would create too long a reference list and here we are not users of these pieces of software but discussing them as pieces of data.

> Reviewer #6: As a new reviewer of this paper, I took time to reflect on the manuscript and the reviewer reports and responses. I agree with the other reviewers that the true core software libraries in scientific computing are things like BLAS, which are not covered by the data scope of the paper. At the same time I agree with the authors that their analysis nevertheless has value because it scopes a method and applies it to demonstrated networked software dependencies and risks within a narrower field of science. It contributes because it shows how immense value and interdependency emerges from the decentralized work of scientists. We should care about these libraries because, as the authors point out, incentives and of the maintainers in this part of the software stack is different from incentives in OSS coding in general.

We appreciate this confidence in the usefulness of the analysis.

> Reviewer #7: I applaud the authors' efforts to improve this manuscript in previous rounds. As a new reviewer added just to this round, I don't think I have too many major comments, except that as a user of the CZI dataset, I have deep concerns of using this dataset for quantitative works. In our preliminary examination, we found pretty strong software name disambiguation problem, which was not captured by CZI's evaluation. I

> believe by focusing on just three software ecosystems, this problem can be alleviated, however, it cannot be totally solved. The one example that I hope the authors could check is Prism, which is a popular software name but is very poorly disambiguated in this dataset. So while the authors acknowledged this as a limitation, in my opinion, this is more than just a limitation that the authors should consider before designing this research.

The disambiguation issue concerns the disambiguation issues inherent in probabilistic datasets such as these. We do address these in the paper as a limitation, and also want to highlight R7's point about their impact being less in the 3 ecosystems we study. Nonetheless, we agree that this is an issue. Our perspective is that as a problem, disambiguation will never be truly solved, but we believe our results can be robust to the issue and our analyses can be repeated straightforwardly from our public codebase if updated datasets become available in the future. We note that Reviewer #6 took the time to make that point themselves, so we feel confident it is acceptable. Accordingly, we addressed this point on page 13 (at the end of disambiguation limitations discussion), writing,

"Nonetheless, we think that this contribution is useful, both as a demonstration of this approach and because our analysis is reproducible. This means that limitations can be addressed in improvements over time. For example, as datasets emerge with improvements to disambiguation these can be incorporated into the analysis."

> A related comment is that, in my opinion, some more basic software, such as scipy and numpy, are way less frequently mentioned than they should be. This is an inevitable bias in scientific methods reporting: not every actually used software will be reported in the scientific papers. I hope the authors could reflect on this bias and talk about alternative ways to trace the impact of software (such as through citations) if they see fit.

We agree that there are many reasons that some packages are mentioned more than others. One of the core ones is that important dependencies of software packages are less likely to be named. We hope that the method of the paper whereby we use the transitive dependencies will help in assisting analyses to acknowledge and ameliorate this issue. In the end we did not make specific edits for this as we hope the introduction makes this issue clear.
